# Significance of CO_2 _donor on the production of succinic acid by *Actinobacillus succinogenes *ATCC 55618

**DOI:** 10.1186/1475-2859-10-87

**Published:** 2011-10-31

**Authors:** Wei Zou, Li-Wen Zhu, Hong-Mei Li, Ya-Jie Tang

**Affiliations:** 1 Key Laboratory of Fermentation Engineering (Ministry of Education), Hubei University of Technology, Wuhan 430068, China; 2 Laboratory of Synthetic Biology, Shanghai Advanced Research Institute, Chinese Academy of Sciences, Shanghai 201203, China; 3 National Key Laboratory of Biochemical Engineering, Institute of Process Engineering, Chinese Academy of Sciences, Beijing 100080, China; 4 State Key Laboratory of Bioreactor Engineering, East China University of Science and Technology, 130 Meilong Road, Shanghai 200237, China

## Abstract

**Background:**

Succinic acid is a building-block chemical which could be used as the precursor of many industrial products. The dissolved CO_2 _concentration in the fermentation broth could strongly regulate the metabolic flux of carbon and the activity of phosphoenolpyruvate (PEP) carboxykinase, which are the important committed steps for the biosynthesis of succinic acid by *Actinobacillus succinogenes*. Previous reports showed that succinic acid production could be promoted by regulating the supply of CO_2 _donor in the fermentation broth. Therefore, the effects of dissolved CO_2 _concentration and MgCO_3 _on the fermentation process should be investigated. In this article, we studied the impacts of gaseous CO_2 _partial pressure, dissolved CO_2 _concentration, and the addition amount of MgCO_3 _on succinic acid production by *Actinobacillus succinogenes *ATCC 55618. We also demonstrated that gaseous CO_2 _could be removed when MgCO_3 _was fully supplied.

**Results:**

An effective CO_2 _quantitative mathematical model was developed to calculate the dissolved CO_2 _concentration in the fermentation broth. The highest succinic acid production of 61.92 g/L was obtained at 159.22 mM dissolved CO_2 _concentration, which was supplied by 40 g/L MgCO_3 _at the CO_2 _partial pressure of 101.33 kPa. When MgCO_3 _was used as the only CO_2 _donor, a maximal succinic acid production of 56.1 g/L was obtained, which was just decreased by 7.03% compared with that obtained under the supply of gaseous CO_2 _and MgCO_3_.

**Conclusions:**

Besides the high dissolved CO_2 _concentration, the excessive addition of MgCO_3 _was beneficial to promote the succinic acid synthesis. This was the first report investigating the replaceable of gaseous CO2 in the fermentation of succinic acid. The results obtained in this study may be useful for reducing the cost of succinic acid fermentation process.

## Background

Succinic acid, an intermediate in the cycle of tricarboxylic acid (TCA), is one of four-carbon platform chemicals for producing different kinds of petroleum derivatives and biodegradable polymers [1,2]. Succinic acid could be produced by chemical conversion and microbial fermentation [3]. Because of the rising price, the limited reserves of petroleum and the pollution of environment, the oil-based industries had been prompted a movement towards the bio-based chemicals, and the bio-based succinic acid production had drawn the attention from enterprises and research institutes [4,5].

As the end-product of the energy metabolism, succinic acid could be produced by many anaerobic microbes, such as *Actinobacillus succinogenes, Anaerobiospirillum succiniciproducens, Mannheimia succiniciproducens, Escherichia coli*, and other microbes [2,4,6,7]. Especially *A. succinogenes *ATCC 55618, which is a facultative anaerobe isolated from the bovine rumen [8]. In the production of succinic acid by *A. succinogenes*, one of the key factors is the supply of CO_2_. A higher concentration of CO_2 _could increase the ratio of succinic acid concentration to the other acids production, the ratio of carbon recovery, and the yield of succinic acid [9]. When *A. succinogenes *and *A. succiniciproducens *were used for the production of succinic acid, as a kind of co-substrate of phosphoenolpyruvate (PEP)-carboxykinase in the TCA cycle, CO_2 _could promote carbon flow toward the production of succinic acid [10,11]. For the other succinic acid production microorganisms such as *E. coli *and *Mannheimia succiniciproducens*, CO_2 _was incorporated into the backbone of three-carbon compound to generate four-carbon oxaloacetate via PEP carboxylase to enhance the production of succinic acid [12,13].

Because of the poor solubility of gaseous CO_2 _at 1 atm, many kinds of carbonate and bicarbonate salts were employed as indirect CO_2 _donor to enhance the dissolved CO_2 _concentration in fermentation broth. MgCO_3 _was a preferable carbonate because the addition of MgCO_3 _would not lead to a radical change of culture pH, and an increase of Mg^2+ ^concentration in fermentation broth showed little negative effect on the metabolism profile and morphology of succinic acid production strain [14]. Some investigators tried to demonstrate the relationship between extra CO_2 _donors and succinic acid production [9,15,16]. But there were a few different features in physiological and biochemical characteristics among various kinds of succinic acid producing strains, and the current results were weak in promoting succinic acid production [15,16].

In this study, the dissolved CO_2 _concentration and the addition amount of MgCO_3 _were quantitatively determined to optimize succinic production by *Actinobacillus succinogenes *ATCC 55618. To calculate the dissolved CO_2 _concentration in the fermentation broth, a mathematical model which considers culture pH, temperature, ionic strength, and salt concentration in the fermentation broth were developed. According to the model prediction and experimental verification, this work firstly demonstrated that the supply of gaseous CO_2 _had no significant effect on succinic acid production when MgCO_3 _was fully supplied.

## Methods

### Maintenance and preculture of *Actinobacillus succinogenes*

The strain of *A. succinogenes *ATCC 55618 was purchased from American Type Culture Collection (ATCC, Manassas USA), which was maintained in 20% glycerol at -70°C.

The plate was inoculated with the above strain and incubated at 37°C for 2 days. Preculture medium consisted of the following components (g/L): tryptone 17; soya peptone 3; glucose 2.5; NaCl 5; K_2_HPO_4 _2.5, and culture pH was adjusted to 7.1-7.5. For the first preculture, 50-mL medium was prepared in a 250-mL anaerobic bottle, and then a colony from a plate culture was inoculated, and followed by 12-hour incubation at 37°C on a rotary shaker at 120 rpm. For the second preculture, 47.5-mL medium was prepared in a 250-mL anaerobic bottle, and inoculated with 2.5-mL first preculture broth, then followed by 12-hour incubation at 37°C on a rotary shaker at 120 rpm.

### Fermentation in the stirred-tank bioreactor

The stirred-tank bioreactor used was a 5.0-L (working volume) BioFlo 110 New Brunswick Scientific (NJ, USA) agitated bioreactor with two six-bladed Rushton impellers (5.9-cm i.d.). The lower impeller was 2.5 cm above the reactor bottom, and the vertical distance between two impellers was 8.5 cm. The reactor was aerated through a ring sparger with a pore size of 1.0 mm, which was located 2.2 cm above the reactor bottom. The bioreactor was equipped with probes of pH (Mettler-Toledo GmbH, Switzerland), temperature and foam.

Fermentation medium was composed of (g/L): glucose 100; yeast extract (YE) 16; corn steep liquor (CSL) 12; KH_2_PO_4 _3; NaCl 1; MgCl_2_·6 H_2_O 0.3; and CaCl_2_·2 H_2_O 0.3. Fermentation was conducted at 37°C and inoculated with 5% (v/v) of the second preculture broth. The rotation speed and external CO_2 _gas sparging rate was 200 rpm and 0.1 vvm. The pH was adjusted to 7.5 with 28% (w/w) ammonia solutions.

Four cultures were carried out simultaneously in the stirred-tank bioreactors with homogeneous cell source under well-controlled process conditions but under different culture conditions. The identical cell source and process conditions, other than the experimental condition, made it possible to perform accurate head-to-head comparisons. The results presented here were confirmed to be reproducible in another experiment (data not shown).

### Model description and calculation

When a gas mixture of CO_2 _and N_2 _was supplied into the bioreactor and became liquid-gas phase equilibrium, the CO_2 _dissolved concentration in broth at 1 atm could be described by the reduction of Henry's law:

(1) $$C_{\text{C}{\text{O}}_{\text{2}}}=\frac{\;P_{\text{C}{\text{O}}_{\text{2}}}}{H}$$

Where *P*_CO2 _is the CO_2 _partial pressure (kPa) in gas mixture which is determined by the mixing ratio of CO_2 _and N_2_, *H *is the Henry's constant for CO_2 _in the fermentation broth (kPa·L/mol), and *C*_CO2 _is the dissolved CO_2 _concentration in the fermentation broth (mol/L).

Since a culture medium contains different kinds of salts and organic substances, the solubility of CO_2 _was described according to an empirical model suggested by Rischbieter et al. [17], and Weisenberger and Schumpe [18]:

(2) $$\log\left(\frac{H}{H_{0}}\right)=\log\left(\frac{c_{G,0}}{c_{G}}\right)=\overset{}{\underset{i}{\sum }}\left(h_{i}+h_{G}\right)c_{i}+\overset{}{\underset{j}{\sum }}\left(b_{n}+b_{G}\right)c_{n,j}$$

Where *H_0 _*is the Henry's constant for CO_2 _in the pure water (kPa·L/mol), *c_i _*is the concentration of ion *i *(mol/L), *c_n, j _*is the concentration of organic substance *j *(g/L). *c_G, 0 _*and *c_G _*denote the gas solubility in pure water and fermentation broth. *h_i _*indicated ion-specific parameter (L/mol), and the *h_i _*values are: Na^+^: 0.1143; Ca^2+^: 0.1762; Mg^2+^: 0.1694; K^+^: 0.0922; Cl^-^: 0.0318; H_2_PO_4_^-^: 0.0906; H^+^: 0; OH^-^: 0.0839; HCO_3_^-^: 0.0976; CO_3_^2-^: 0.1423. *b_n _*indicates substance-specific model parameter (m^3^/kg), and the *b_n _*for glucose, YE and CSL are: 6.68 × 10^-4 ^m^3 ^/kg, 7.9 × 10^-4 ^m^3 ^/kg, and 2.11 × 10^-4 ^m^3 ^/kg. *h_G _*was estimated by Equation (3) following the suggestions of Weisenberger and Schumpe [18]:

(3) $$h_{G}=h_{G,0}+h_{G,T}\left(T-298.15K\right)$$

where *h_G, 0 _*and *h_G, T _*for CO_2 _are -0.0172 L/mol and -0.338 × 10^-3 ^L/mol·K, respectively, between 273-313 K [18]. And *b_G _*in Equation (2) was estimated by Equation (4) as suggested by Rischbieter et al. [17]:

(4) $$b_{G}=b_{G,0}+b_{G,T}\left(T-298.15K\right)$$

where *b_G, 0 _*and *b_G, T _*for CO_2 _are -1.86 × 10^-4 ^m^3^/kg and 0.01 × 10^-4 ^m^3^/kg·K, respectively, between 283-303 K [17]. Combining Equations (2), (3) and (4), a model of Henry's constant for CO_2 _in fermentation medium could be obtained.

(5) $$\log(\frac{H}{H_{0}})=\sum_{i}(h_{i}+h_{G,0}+h_{G,T}(T-298.15K))c_{i}+\sum_{j}(b_{n}+b_{G,0}+b_{G,T}(T-298.15K))c_{n,j}$$

In Equation (5), the Henry's constant of CO_2 _in the pure water was 4320 kPa L/mol [15]. *T *is the absolute temperature (K) in culture condition.

**Figure 1:**
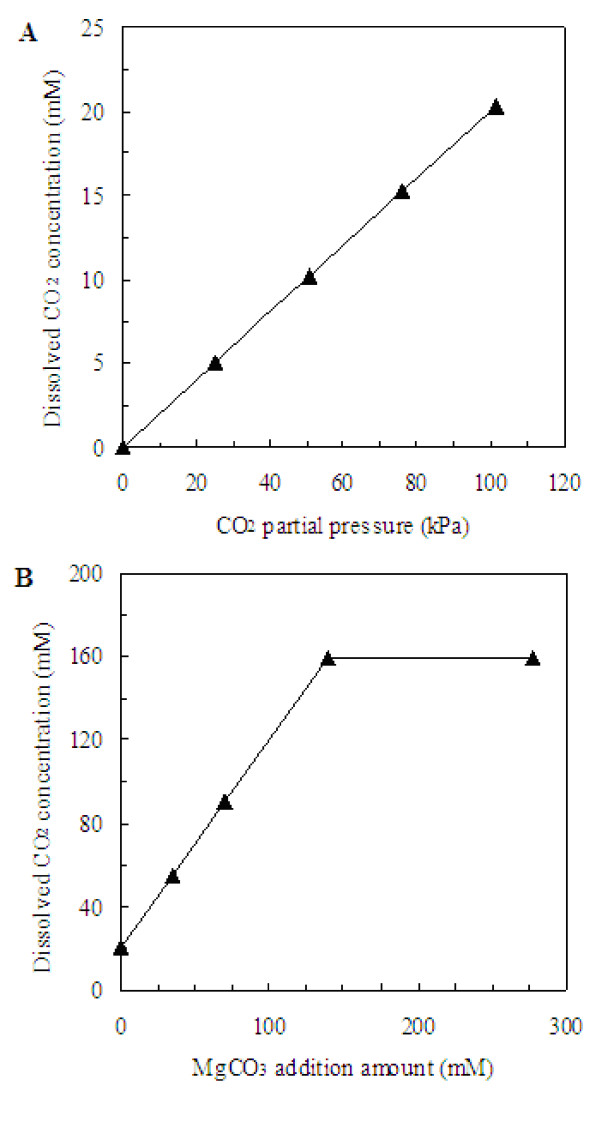
**Effects of gaseous CO_2 _and MgCO_3 _on the dissolved CO_2 _concentration in the fermentation broth**. (A): The dissolved CO_2 _concentration in the fermentation broth with the sole supply of various CO_2 _partial pressures. (B): The dissolved CO_2 _concentration in the fermentation broth with the addition of various amount of MgCO_3 _at the CO_2 _partial pressure of 101.33 kPa (i.e., 100% CO_2 _gas).

After combining all the parameters mentioned above into the Equation (5) and Equation (1), the model used for calculating CO_2 _dissolved concentration in broth could be obtained when gaseous CO_2 _was used as external CO_2 _donor and the result is shown in Figure 1A. There is a correlated linear trend between CO_2 _partial pressure and the dissolved CO_2 _concentration in fermentation broth. The dissolved CO_2 _concentration in the fermentation broth was 5.05, 10.11, 15.16 and 20.22 mM when CO_2 _partial pressure was 25.33, 50.66, 75.99 and 101.33 kPa, respectively. And the maximal dissolved CO_2 _concentration is 20.22 mM due to the solubility of gaseous CO_2_.

When MgCO_3 _was added with the supply of pure gaseous CO_2 _at 1 atm, CO_2_, HCO_3_^-^, and CO_3_^2- ^would become in equilibrium in the fermentation broth according to the following equations [15]:

(6) $$CO_{2}+H_{2}O↔HCO_{3}^{-}+H^{+}$$

(7) $$HCO_{3}^{-}↔CO_{3}^{2-}+H^{+}$$

As reported in the previous study [19], the maximum solubility of MgCO_3 _in water at 40°C was 139 mM. According to Equation (1), (5), (6) and (7), the model used for calculating the dissolved CO_2 _concentration in the fermentation broth could be obtained when both gas phase CO_2 _and MgCO_3 _were used as CO_2 _donors, and the relationship between the addition amount of MgCO_3 _and the dissolved CO_2 _concentration under the CO_2 _partial pressure of 101.33 kPa is shown in Figure 1B. The CO_2 _concentrations in the fermentation broth were 20.22, 54.97, 89.72, 159.22 and 159.22 mM when the addition of MgCO_3 _was 0, 2.92, 5.84, 11.68, and 23.35 g/L, respectively. And the maximum dissolved CO_2 _concentration is 159.22 mM due to the solubility of gaseous CO_2 _and MgCO_3_.

### Effect of CO_2 _partial pressure

The significance of gaseous CO_2 _partial pressure on succinic acid accumulation was studied by setting CO_2 _partial pressure at 25.33, 50.66, 75.99 and 101.33 kPa during the whole fermentation process in the stirred-tank bioreactors, which was controlled by adjusting the corresponding mixing ratio of CO_2 _and N_2 _at 25%, 50%, 75%, 100% (v: v) by gas mix controller (BioFlo110, New Brunswick Scientic NJ, USA), respectively, and the corresponding dissolved CO_2 _concentration in the fermentation broth was 5.05, 10.11, 15.16, and 20.22 mM.

### Effect of the supply of gaseous CO_2 _and the addition of MgCO_3_

The maximal dissolved CO_2 _concentration in the fermentation broth was 20.22 and 139.00 mM when only gaseous CO_2 _and MgCO_3 _was supplied, respectively. In order to study the higher dissolved CO_2 _concentration on the succinic acid production, the fermentations were conducted by adding MgCO_3 _to enhance the dissolved CO_2 _concentration. MgCO_3 _was added to the broth after a separate sterilization before the inoculation. The effect of the supply of gaseous CO_2 _and the addition of MgCO_3 _on the fermentation process was studied by adding 2.92, 5.84, 11.68, and 23.35 g/L of MgCO_3 _at the CO_2 _partial pressure of 101.33 kPa (i.e., 100% CO_2 _gas), and its corresponding dissolved CO_2 _concentrations in the fermentation broth were 54.97, 89.72, 159.22 and 159.22 mM, respectively. The dissolved CO_2 _concentration maintained constant at 159.22 mM even when concentrations higher than 11.68 g/L of MgCO_3 _were added at the CO_2 _partial pressure of 101.33 kPa. The other culture conditions were the same as the above experiments.

### Effect of the addition of higher amount of MgCO_3_

Effect of the excessive addition amount of MgCO_3 _was studied by adding 30, 40, 50 and 60 g/L of MgCO_3 _at the CO_2 _partial pressure of 101.33 kPa (i.e., 100% CO_2 _gas), and all the corresponding dissolved CO_2 _concentration in the fermentation broth was 159.22 mM. The other culture conditions were the same as the above experiments.

### Effect of CO_2 _donor supply mode

According to the above results and Equation (5), the effect of CO_2 _donor supply mode was studied by using two supply modes: 40 g/L MgCO_3 _was used as the only CO_2 _donor, and 40 g/L MgCO_3 _was supplied at the CO_2 _partial pressure of 101.33 kPa (i.e., 100% CO_2 _gas). The other culture conditions were the same as the above experiments.

### Sampling, the determination of succinic acid production

For sampling, about 20-30 mL of broth was taken once from each reactor and the cell growth was monitored by measuring the optical density at 660 nm (OD_660_). At an OD_660 _of 1.0, *A. succinogenes *ATCC 55618 has a concentration of 0.626 g dry cell weight (DCW)/L. For succinic acid determination, 1 mL methanol and 1 mL acetonitrile were added to 1 mL fermentation broth to remove protein and the sample was kept at 4°C overnight. After centrifuging at 11, 000 rpm for 30 min, the supernatants were diluted and filtrated through 0.22 μm filter, then analyzed by high-performance liquid chromatography (HPLC, Dionex) using Maisch ReproSil-Pur Basic C18 column. The optimized mobile phase was 5 mM KH_2_PO_4 _water solution, whose pH was adjusted to 2.8 by H_3_PO_4_. The column oven temperature was maintained at 40°C and the flow rate was 1 mL/min. The detection wave was 210 nm. Residual sugar level was assayed with phenol-sulfuric acid method [20].

## Results and discussion

### Effect of CO_2 _partial pressure

As one of the direct substrates for the biosynthesis of succinic acid, CO_2 _could affect the metabolic flux and the mass distribution of succinic acid [8,21]. The quantitative determination of the dissolved CO_2 _concentration in the fermentation broth is beneficial to study the impact of CO_2 _partial pressure on the production of succinic acid. Song et al. [15] and Lee et al. [16] reported that succinic acid production could be enhanced by increasing CO_2 _partial pressure in the fermentation of *M. succiniciproducens *and *A. succiniciproducens*. Therefore, it was necessary to investigate the effect of CO_2 _partial pressure on the accumulation of succinic acid by *A. succinogenes *ATCC 55618.

**Figure 2:**
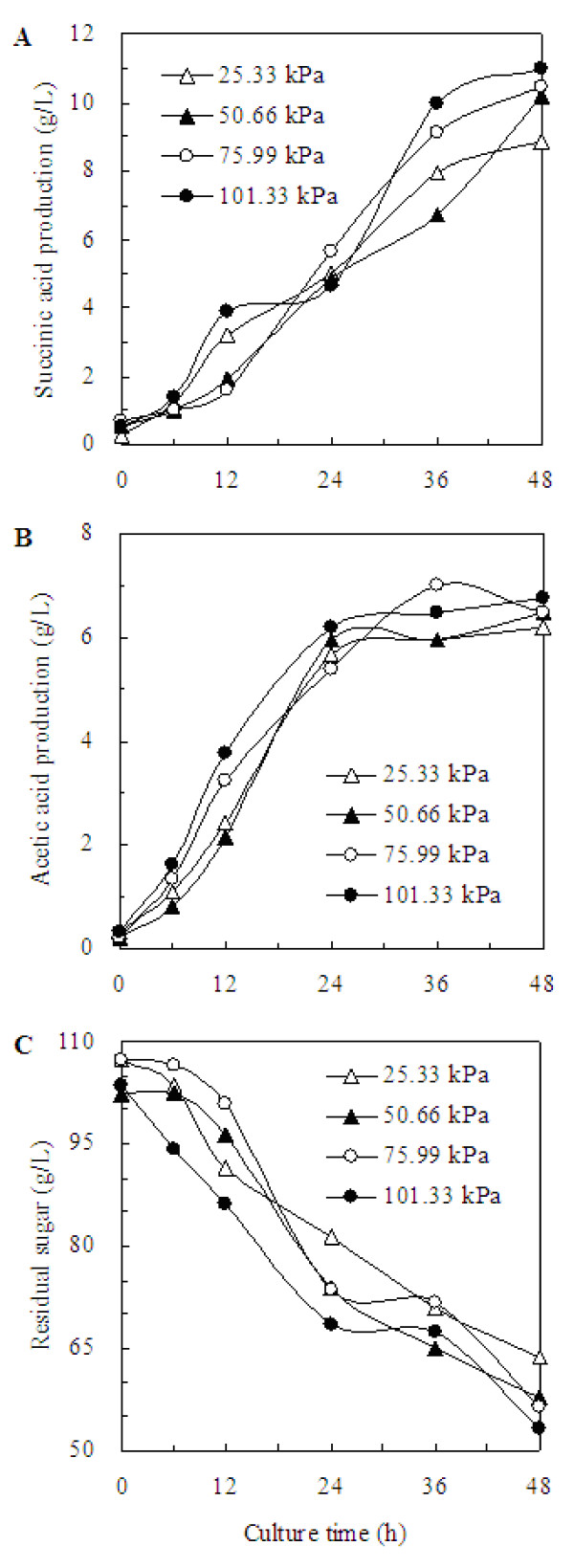
**Effect of CO_2 _partial pressure on the succinic acid production during the *A. succinogenes *fermentation**. (A) the succinic acid production, (B) the acetic acid production, (C) the sugar consumption.

The effect of CO_2 _partial pressure on the succinic acid production is shown in Figure 2A. When the CO_2 _partial pressures were 25.33, 50.66, 75.99, and 101.33 kPa, the dissolved CO_2 _concentrations in the fermentation broth calculated using Equation (5) was 5.05, 10.11, 15.16, and 20.22 mM, respectively (Table 1). And at the CO_2 _partial pressure of 101.33 kPa, the maximal dissolved CO_2 _concentration achieved was 20.22 mM, which was the highest dissolved CO_2 _concentration when only gaseous CO_2 _was supplied. The dissolved CO_2 _concentration was increased with the increase of the partial pressure when gaseous CO_2 _was used as sole CO_2 _donor. The succinic acid productions were 8.84, 10.21, 10.44, and 10.97 g/L as obtained on 48 hour at the CO_2 _partial pressure of 25.33, 50.66, 75.99, and 101.33 kPa, respectively, and its corresponding productivities were 0.18, 0.21, 0.22, and 0.23 g/L per hour. This indicated that when gaseous CO_2 _was used as the sole CO_2 _donor, CO_2 _partial pressure showed no significant effect on the succinic acid accumulation. On the contrary, as reported by Lu et al. [22] and Samuelov et al. [23], a higher available CO_2 _concentration could cause higher succinic acid production by increasing the activity of PEP carboxykinase. These indicated that when gaseous CO_2 _was used as the sole CO_2 _donor, the available dissolved CO_2 _concentration was not high enough to increase the production of succinic acid in the fermentation of *A. succinogenes*.

**Table 1:** Effect of CO_2 _donor on the growth of *A.succinogenes *and succinic acid production parameters

Addition amount of MgCO_3_ (g/L)	Gaseous CO_2 _partial pressure (kPa)^a^	Dissolved CO_2 _concentration (mM)	OD_660_	Succinic acid productivity (g/L per hour)	Specific succinic acid productivity (g succinic acid/g DCW)	Yield of succinic acid against glucose (g succinic acid/g glucose)
-^a^	25.33	5.05	6.61	0.18	3.50	0.19
-	50.66	10.11	6.69	0.21	3.23	0.22
-	75.99	15.16	6.51	0.22	3.10	0.19
-	101.33	20.22	6.03	0.23	3.57	0.21
2.92	101.33	54.97	10.12	0.32	2.71	0.27
5.84	101.33	89.72	10.27	0.33	2.93	0.26
11.68	101.33	159.22	9.72	0.54	5.13	0.38
23.35	101.33	159.22	10.94	0.77	6.53	0.45
30	101.33	159.22	9.10	0.74	11.04	0.56
40	101.33	159.22	9.62	0.86	13.60	0.60
50	101.33	159.22	9.74	0.85	12.66	0.64
60	101.33	159.22	9.62	0.81	11.44	0.63
40	101.33	159.22	10.31	0.84	12.02	0.58
40	-	139.00	9.65	0.80	11.77	0.54

^a ^Gas mixture was composed of CO_2 _and N_2_.

^b ^- means the CO_2 _donor was not add.

As shown in Figure 2B, the patterns of acetic acid production at various CO_2 _partial pressures were similar. The concentrations of other by-products such as formic acid, lactic acid and ethanol were relatively constant at around 5.0, 11.0 and 2.0 g/L, respectively, regardless of the levels of the dissolved CO_2 _in the broth.

Figure 2C shows the time profile of residual sugar under various CO_2 _partial pressures. The glucose concentration at the CO_2 _partial pressure of 101.33 kPa was decreased faster than that at other CO_2 _partial pressures during the first 24 hours. The yield of succinic acid against glucose was around 0.21 g succinic acid/g glucose when gaseous CO_2 _was used. That means the CO_2 _partial pressure showed no significant effect on the succinic acid yield. And there was no significant effect on the cell growth. The OD_660 _was between 6.0 and 6.7 when gaseous CO_2 _partial pressure was 25.33, 50.66, 75.99, and 101.33 kPa (Table 1).

### Effect of the supply of gaseous CO_2 _and the addition of MgCO_3_

The maximal dissolved CO_2 _concentration is limited by the solubility of gaseous CO_2 _when it was supplied as the sole CO_2 _donor. In order to investigate the effect of higher dissolved CO_2 _concentration on succinic acid production, the fermentations were conducted by adding MgCO_3 _at the CO_2 _partial pressure of 101.33 kPa (i.e., 100% CO_2 _gas) to enhance the dissolved CO_2 _concentration in the fermentation broth.

**Figure 3:**
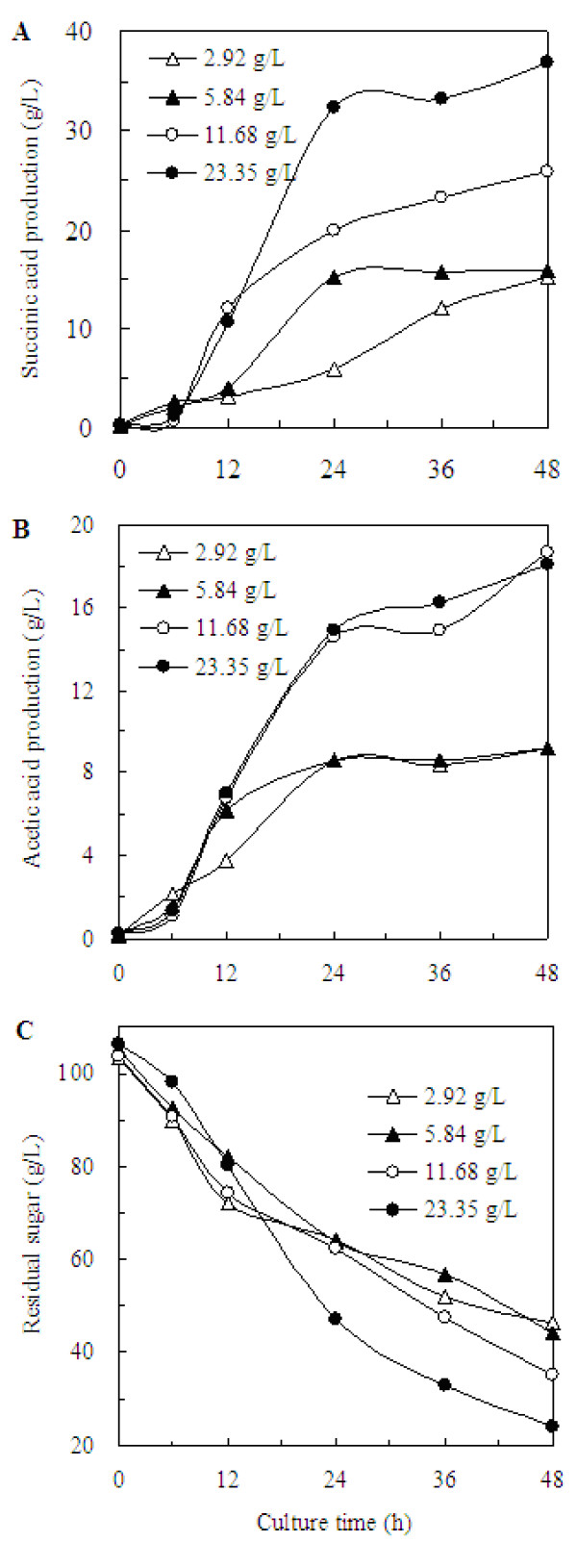
**Effect of the supply of gaseous CO_2 _and the addition of MgCO_3_**. (A) the succinic acid production, (B) the acetic acid production, (C) the sugar consumption. The fermentation was performed at the CO_2 _partial pressure of 101.33 kPa.

The effect of the supply of gaseous CO_2 _and the addition of MgCO_3 _on the fermentation process was studied by adding 2.92, 5.84, 11.68, and 23.35 g/L of MgCO_3 _at the CO_2 _partial pressure of 101.33 kPa (i.e., 100% CO_2 _gas), and the corresponding dissolved CO_2 _concentrations was 54.97, 89.72, 159.22, and 159.22 mM. Because of the solubility of MgCO_3 _and CO_2_, the maximal dissolved CO_2 _concentration of 159.22 mM was obtained under the addition of 11.68 g/L MgCO_3 _with 100% CO_2 _gas. Even more than 11.68 g/L of MgCO_3_, the dissolved CO_2 _concentration maintained constant at 159.22 mM. As shown in Figure 3A, the highest succinic acid productions were 15.26, 15.94, 25.86 and 36.84 g/L as obtained on 48 hour with the addition of 2.92, 5.84, 11.68 and 23.35 g/L MgCO_3_, respectively, and its corresponding productivity was 0.32, 0.33, 0.54 and 0.77 g/L per hour, respectively. The maximum succinic acid production was increased from 25.86 to 36.84 g/L when the addition amount of MgCO_3 _was increased from 11.68 to 23.35 g/L, while the dissolved CO_2 _concentration was maintained constant. It can be concluded that the higher dissolved CO_2 _concentration was beneficial for the succinic acid biosynthesis. But the dissolved CO_2 _concentration was not the only factor affecting succinic acid synthesis; the excessive MgCO_3 _also had positive effect. As a kind of neutralization reagent, MgCO_3 _could promptly neutralize the organic acid produced during the fermentation process. But when only 11.68 g/L of MgCO_3 _was added, the saturated state of MgCO_3 _would be lost quickly because of the rapid accumulation of organic acid during the fermentation. And when the addition amounts of MgCO_3 _exceeded 11.68 g/L, there will be excessive solid MgCO_3 _precipitate. Even if organic acids accumulate, MgCO_3 _solution can also keep saturated.

Equation (6) and (7) indicated that the dissolved concentrations of HCO_3_^-^, CO_3_^2- ^and CO_2 _could be enhanced with the addition of MgCO_3 _in the fermentation broth. However, MgCO_3 _may not be used as CO_3_^2- ^donor because there were few reports that CO_3_^2- ^could be used directly as substrate by succinic acid producing microorganisms. Although HCO_3_^- ^and CO_2 _could be used as the co-substrate of PEP carboxylase and improve the production of succinic acid [23], HCO_3_^- ^was much less permeable to lipid cell membrane than the uncharged CO_2 _molecule because is a kind of polar molecular, and there was no HCO_3_^- ^transporter on the membrane of *A. succinogenes *which could deliver HCO_3_^- ^from the broth into the cell [24]. So the higher concentration of HCO_3_^- ^could not promote the production of succinic acid. And MgCO_3 _may be used as indirect CO_2 _molecule donor to promote the production of succinic acid in the fermentation process of *A. succinogenes*. On the other hand, when the levels of dissolved CO_2 _reached 159.22 mM, there would be insoluble MgCO_3_, and that could cause turbid broth. The cells were spread uniformly in the broth, which was helpful to eliminate the cell flocculation and indirectly promoting the succinic acid biosynthesis.

As shown in Figure 3B, the patterns of acetic acid production at the dissolved CO_2 _concentration of 54.97 and 89.72 mM were similar. However, when the levels of dissolved CO_2 _reached 159.22 mM, the acetic acid production was significantly enhanced. It was distinct from other reports. In the fermentation of *M. succiniciproducens*, the levels of dissolved CO_2 _showed little effects on the acetic acid accumulation [15]. The CO_2 _concentration has been shown to regulate the levels PEP carboxykinase pathway at high CO_2 _levels, and PEP carboxykinase levels rise [23]. However, the enhanced PEP carboxylation may cause higher glucose consumption rate. This effect may cause more metabolic flow by PEP to pyruvic acid, and further to acetic acid. Meanwhile the production of formic acid, lactic acid and ethanol almost not be improved may be because the raised PEP carboxykinase activity competitively inhibited these key enzymes such as pyruvate formatelyase, lactate dehydrogenase and ethanol dehydrogenase.

The patterns of cell growth with the addition of different concentration of MgCO_3 _were similar, and the OD_660 _was between 9.7 and 10.9 (Table 1). Figure 3C shows the time profile of residual sugar under various addition amount of MgCO_3_. When 23.35 g/L of MgCO_3 _was added, glucose was consumed faster than the other conditions between 12 and 24 hour, which corresponded well to the succinic acid accumulation. The yield of succinic acid against glucose was 0.27, 0.26, 0.38, and 0.45 g succinic acid/g glucose when the addition amount of MgCO_3 _was 2.92, 5.84, 11.68 and 23.35 g/L, respectively. This indicated that the higher dissolved CO_2 _concentration could effectively improve the yield of succinic acid against glucose.

### Effect of the addition of higher amount of MgCO_3_

Effect of the higher addition amount of MgCO_3 _was studied by adding 30, 40, 50 and 60 g/L of MgCO_3 _at the CO_2 _partial pressure of 101.33 kPa (i.e., 100% CO_2 _gas), and all the corresponding dissolved CO_2 _concentration in the fermentation broth were 159.22 mM.

**Figure 4:**
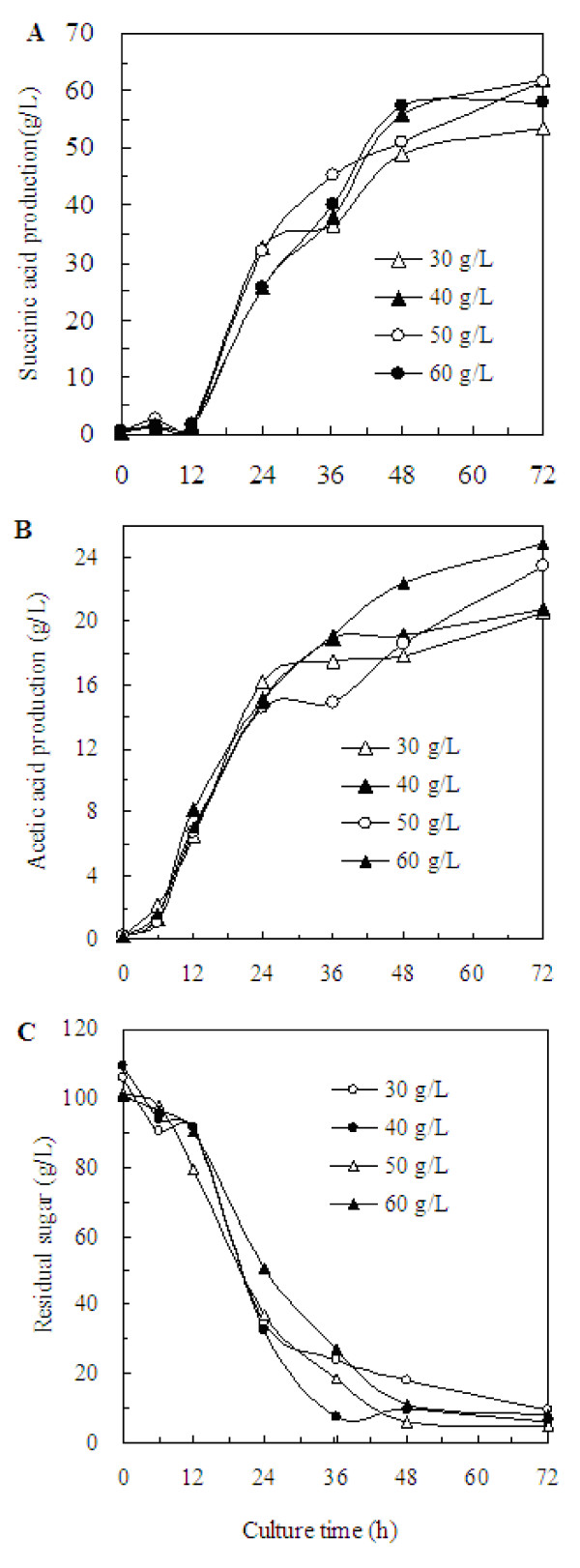
**Effect of the excessive addition amount of MgCO_3 _on the succinic acid production**. (A) the succinic acid production, (B) the acetic acid production, (C) the sugar consumption.

As shown in Figure 4A, the pattern of succinic acid production under various addition amount of MgCO_3 _within the range of investigation was similar. The maximal succinic acid production of 53.55, 61.92, 61.48, and 58.05 g/L was obtained with the addition of 30, 40, 50, and 60 g/L MgCO_3_, respectively, and its corresponding productivity was 0.74, 0.86, 0.85 and 0.81 g/L per hour. When the addition amount of MgCO_3 _exceeded 40 g/L, the production and productivity of succinic acid were kept almost constant, but the specific productivity was decreased. This indicated 40 g/L MgCO_3 _was enough for improving the accumulation of succinic acid. Similarly, Du et al. [25] reported when the addition amount of MgCO_3 _exceeded 30 g/L, there was no significant change on the production of succinic acid.

The significance of addition amount of MgCO_3 _on acetic acid accumulation was studied. As shown in Figure 4B, there was no significant effect on the biosynthesis of acetic acid. Similarly, the concentrations of other by-products such as formic acid, lactic acid and ethanol were relatively constant at around 5.0, 11.0 and 2.0 g/L, respectively, regardless of the addition amount of MgCO_3_.

The cell growth patterns under the addition of MgCO_3 _were quite similar (Table 1). Figure 4C shows that the time profile of residual sugar under various addition amount of MgCO_3_. The yield of succinic acid against glucose was 0.56, 0.60, 0.64, and 0.63 g succinic acid/g glucose when the addition amount of MgCO_3 _was 30, 40, 50 and 60 g/L, respectively. It seemed that to obtain a higher yield of succinic acid against glucose, the addition amount of MgCO_3 _should no be more than 50 g/L.

### Effect of CO_2 _donor supply mode

Gaseous CO_2 _was widely used as external CO_2 _donor and anaerobic environment maintenance agent in succinic acid fermentation process. Calculated from Equation (5, 6, 7), the dissolved CO_2 _concentration (139.00 mM) under 40 g/L MgCO_3 _was just decreased by 12.76% comparing with that obtained under the addition of 40 g/L MgCO_3 _with 100% CO_2 _gas. This suggested that the gaseous CO_2 _may be removed by the addition of MgCO_3_. In order to testify this proposal, the effect of CO_2 _donor supply mode was studied by using two supply modes: 40 g/L MgCO_3 _was used alone; 40 g/L MgCO_3 _was supplied with 100% CO_2 _gas.

**Figure 5:**
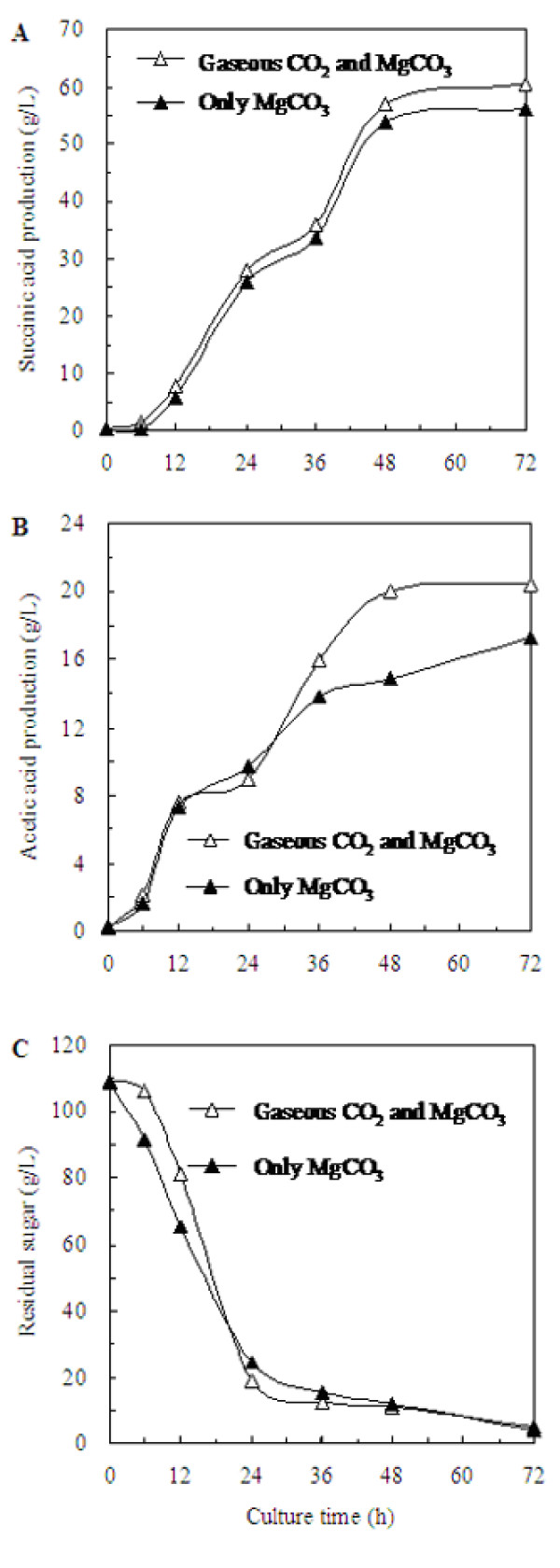
**Effect of CO_2 _donor supply mode on succinic acid production during the *A. succinogenes *fermentation**. (A) the succinic acid production, (B) the acetic acid production, (C) the sugar consumption.

As shown in Figure 5A, after 72 h incubation, the production of succinic acid reached 56.14 and 60.38 g/L when 40 g/L MgCO_3 _was used as the only CO_2 _donor and 40 g/L MgCO_3 _was supplied at the CO_2 _partial pressure of 101.33 kPa, and the corresponding productivity was 0.80 and 0.84 g/L per hour. The succinic acid production was just decreased by 7.03% without the supply of gaseous CO_2_. As shown in Figure 5B, the acetic acid production was decreased by 17.91% without the supply of gaseous CO_2_. Figure 5C clearly shows the time courses of sugar consumption under different CO_2 _supply modes were similar. The yield of succinic acid against glucose was 0.54 g succinic acid/g glucose when 40 g/L MgCO_3 _was used alone, and the yield was 0.58 g succinic acid/g glucose when MgCO_3 _was supplied with 100% CO_2_. And there was no significant effect on the cell growth whether gaseous CO_2 _was used.

## Conclusions

In this study, an effective CO_2 _quantitative mathematical model was developed to calculate the dissolved CO_2 _concentration in the broth during the fermentation of *Actinobacillus succinogenes *ATCC 55618. The model offered a quantitative method for screening the suitable CO_2 _donor form and addition amount for the production of succinic acid. There was no significant effect of CO_2 _partial pressure on the production of succinic acid when gaseous CO_2 _was used as the sole CO_2 _donor. But when gaseous CO_2 _was used with MgCO_3_, higher amount of MgCO_3 _was more effective on promoting the succinic acid synthesis. And the maximum succinic acid production of 61.92 g/L was obtained at 159.22 mM dissolved CO_2 _concentration, which was supplied by 40 g/L MgCO_3 _with 100% CO_2 _gas. And it was concluded that the supply of gaseous CO2 was not essential when 40 g/L of MgCO_3 _was added in the fermentation medium. This is the first report investigating the replaceable of gaseous CO2 in the fermentation of succinic acid. The results obtained in this study may be useful for reducing the cost of succinic acid fermentation process.

## Competing interests

The authors declare that they have no competing interests.

## Authors' contributions

YJT and WZ designed the experiments, WZ carried out the experimental work, LWZ and WZ analyzed data, WZ drafted the manuscript, HML provided some critical discussions and support for this project.

YJT and LWZ critically reviewed and modified the paper. All authors approved the final manuscript.
